# Effects of Using Silica Fume and Polycarboxylate-Type Superplasticizer on Physical Properties of Cementitious Grout Mixtures for Semiflexible Pavement Surfacing

**DOI:** 10.1155/2014/596364

**Published:** 2014-01-02

**Authors:** Suhana Koting, Mohamed Rehan Karim, Hilmi Mahmud, Nuha S. Mashaan, Mohd Rasdan Ibrahim, Herdayati Katman, Nadiah Md Husain

**Affiliations:** ^1^Centre for Transportation Research, Faculty of Engineering, University of Malaya, 50603 Kuala Lumpur, Malaysia; ^2^Universiti Tenaga Nasional, Putrajaya Campus, Jalan Ikram-Uniten, 43000 Kajang, Selangor, Malaysia

## Abstract

Semi-flexible pavement surfacing is a composite pavement that utilizes the porous pavement structure of the flexible bituminous pavement, which is subsequently grouted with appropriate cementitious materials. This study aims to investigate the compressive strength, flexural strength, and workability performance of cementitious grout. The grout mixtures are designed to achieve high strength and maintain flow properties in order to allow the cement slurries to infiltrate easily through unfilled compacted skeletons. A paired-sample *t*-test was carried out to find out whether water/cement ratio, SP percentages, and use of silica fume influence the cementitious grout performance. The findings showed that the replacement of 5% silica fume with an adequate amount of superplasticizer and water/cement ratio was beneficial in improving the properties of the cementitious grout.

## 1. Introduction

To fulfil the demands for better performance in highway construction, various research studies are being carried out. These research and development programs are concentrating on finding better and more effective types of pavement that will provide and satisfy the needs of users. The quality of the road, cost effectiveness, and performance of the pavement are some of the important criteria that have been taken into consideration in order to find the right choice in the design of pavements [1]. Generally, road pavements are categorized into two broad classes as (1) rigid and (2) flexible pavements. The flexible pavement is defined as a pavement with a bituminous surfacing and with a base layer with or without a hydrocarbon binder [2]. The rigid pavement is defined as a pavement substantially constructed of cement concrete [2]. In short, the flexible pavement contains aggregates that are bound by bituminous binders while rigid pavements comprise a variety of cement concrete types with or without reinforcements.

In Malaysia, which is hot and humid throughout the year, the choice of road surfacing is normally a flexible pavement while the rigid pavement is not widely used. This is due to a slow setting time during the construction process of the rigid pavement, poor riding quality, noise problems during usage, and higher costs although the rigid pavements do have a longer durability. The flexible pavement is exposed to greater stresses due to increasing traffic volumes, truck traffic, and higher tire pressures [3]. In Ali et al. [4], it is stated that rutting and fatigue cracking are the major distresses that lead to permanent failures in pavement construction. In addition, diesel spillage issue is also a problem that occurs in conventional bituminous pavement. In order to overcome the disadvantages and improve the performance of both pavements, an alternative semiflexible pavement is considered. Features incorporated into the semiflexible design are a hard surface, antirutting, oil, and heat resistance [1]. In addition to these properties, the pavement must be able to deform without cracking and without joints, thus leading to a lower maintenance cost. In Malaysia, semiflexible pavements have been introduced on public roads especially in areas with heavy traffic (bus lanes, bus and car terminals, heavily trafficked roads, etc.) in order to enhance the road performance and effectiveness, thus offering joint-free and high friction pavements. The semiflexible pavement is designed to withstand the stresses from the wear and tear from heavy continuous traffic, rutting from continual driving along the same routes and sudden braking [5]. As a joint-free pavement, it renders good friction and provides safety and driving comfort [5]. It has been proven that this type of pavement is superior to conventional design pavements according to a number of criteria. The semiflexible pavements will provide longer reliability in terms of its load and rutting resistant performance due to the number of repetition loads that are applied to the pavement daily. The semiflexible pavements have a high indirect stiffness modulus even though the pavements have to carry low loading frequencies. The semiflexible pavement needs less maintenance and repair works. Although the preparation of semiflexible pavement is costly in the early stage of its production, in the long term, this type of pavement is more economical than the conventional flexible pavement.

Although the semiflexible pavement design has been used in Malaysia, it still needs some modifications in terms of design and materials used in order to increase its road performance and effectiveness. The modifications in the semiflexible design are dependent on the climate and also on the thermal expansion that might occur in the pavement. The most important criterion of the semiflexible pavement surfacing is to produce cementitious grouts that achieve sufficient strength and workability.

## 2. Materials and Experimental Program

There are three stages in the production of a semiflexible pavement surfacing. The first stage is to produce acceptable proportions of cementitious grout mixtures. The second stage is to produce unfilled compacted skeletons. The third stage is to produce the cement-bitumen composites (semiflexible pavement surfacing). The development of cementitious grout mixtures is focused here.

### 2.1. Cementitious Grout Mixtures

The main materials used in cementitious grout mixtures are ordinary Portland cement (OPC) and silica fume (SF). The specific gravity of OPC is 3.10 g/cm^3^ and the surface area is 335 m^2^/kg. The specific gravity of silica fume (SF) is 2.2 g/cm^3^ and the surface area is 20000 m^2^/kg.

Water/cement ratio was varied from 0.30 to 0.50. A superplasticizer (SP) with polycarboxylic ether polymer (PCE) chemical based, namely, Viscocrete 25 MP supplied by Sika Services AG, was used in the range of 0.5%–2.5% of cement weight. It meets the requirements for high range water reducing super plasticizers (SP) according to EN 934-2 [6]. This SP was used in liquid form, which was found to be more rapidly dispersed in a uniform way during the mixing of the slurries and therefore increased the workability of the grout mixtures.

### 2.2. Mix Proportions

The cementitious grout mixtures were designed in two main groups, defined as GM1 and GM2. The first cement grout group (GM1) contains 100% of OPC and the second cementitious grout group (GM2a and GM2b) contains about 5% to 10% of silica fume as a replacement to OPC. The mix details of cementitious grout mixtures are shown in Table 1.

In this study, the use of SF was considered to define the influence of the SF content on the workability and the compressive strength performance. The mixture proportions which produce sufficient workability and strength performance were selected to be applied in the semiflexible pavement surfacing prototypes.

### 2.3. Preparation of Cementitious Grout

Cementitious grouts were mixed in the laboratory using 8 liters mechanical mixer with varying speed, in accordance to the ASTM C 305-99: Standard Practice for Mechanical Mixing of Hydraulic Cement Pastes and Mortars of Plastic Consistency [7]. A modified method to that practiced by Zoorob et al. [8] was adopted. At first, all the dry materials were mixed dry for about 1 minute or more depending on the mixture proportions. Half of the mixing water was then added followed by the other half containing the SP. Mixing was continued for further 3 minutes at a higher mixing speed. This mixing procedure was found to minimize the absorption of SP by the binders and therefore improved workability [9]. Flow out time and density tests were conducted on fresh grout mixture while cube compressive strength tests (50 × 50 × 50 mm) and beam flexural strength tests (50 × 50 × 330 mm) were carried out on the hardened concrete.

### 2.4. Laboratory Investigation

In this study, the flow time test was carried out to measure the liquidity of various cementitious grout proportions and to ensure that the grouts have the correct consistency before it penetrates into the unfilled compacted skeletons. The Malaysian Mortar Flow Cone method (City Hall Kuala Lumpur specification (CHKL)) [9] was used in order to examine the workability of cementitious grout mixtures under Malaysian condition. In this test, 1 liter of wet cementitious grout was measured using a cylinder. The high workability cementitious grout was then transferred to a Malaysian flow cone tester. This flow cone is held in a vertical position and the flow cone plug was released while the stop watch was started simultaneously. The flow time was measured by stopping the stopwatch as soon as the flow cone is empty.

The hardened grouts were tested for compressive and flexural strength tests. The compressive strength performance on grout cubes was conducted in 1, 3, 7, and 28 days. The compressive strength test was carried out using an ELE testing machine press with a capacity of 2000 kN and a pacing rate of 0.5 in/s. The flexural strength test was conducted on cementitious grout beams after 7 and 28 days. The flexural strength investigation was carried out for selected mixture proportions, which produced sufficient compressive strength performance.

## 3. Results and Discussions 

### 3.1. Mechanical Properties of Cementitious Grout Mixtures

This section discusses some engineering properties of cementitious grouts, that is, workability, compressive strength, and flexural strength. The grout mixture proportions which exhibited sufficient and acceptable performance will be incorporated later with the unfilled compacted skeletons in order to produce cement-bitumen composites (semiflexible pavement surfacing prototypes).

### 3.2. Workability of Cementitious Grout Mixtures

The quality of concrete can be improved by using suitable materials and appropriate mix proportions [9]. The parameters that affect the quality of concrete include water to cement materials ratio, cement material content, and size and grading of aggregates. Incorporating supplementary cementing materials, such as fly ash, blast furnace slag, and silica fume, can further decrease the permeability of concrete to water and the diffusion of oxygen, chloride, carbon dioxide, and so forth [10].

**Figure 1:**
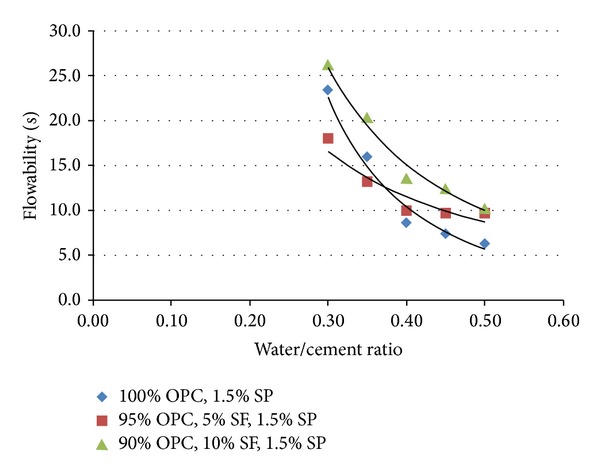
Influence of water/cement ratio on the workability for 100% OPC; 95% OPC, 5% SF; and 90% OPC, 10% SF.

In this study, the parameters discussed are water/cement ratio, percentages of SP, and the use of silica fume as replacement of cement material.  Figure 1 shows the influence of water/cement (w/c) ratio on the workability with different designs of cementitious grout mixtures. The mixture proportions are 100% OPC (GM1); 95% OPC, 5% SF (GM2a); and 90% OPC, 10% SF (GM2b), with respect to 0.30, 0.35, 0.40, 0.45, and 0.50 w/c ratio. The SP percentage was fixed at 1.5% by using a polycarboxylic ether polymer (PCE) type. It was determined from a study that factor affecting workability was the water content of the mix, since by simply adding water the interparticle lubricant is increased. The higher the water/cement ratio is, the lower the viscosity is, because the flow time is inversely proportional to the water content.

It can be seen that there is a reduction in the flow value when increasing the w/c ratio from 0.30 to 0.50. For a w/c ratio of 0.30, the highest flow value is 26.2 s which was produced by 90% OPC, 10% SF. The flow values are constantly reduced until the w/c ratio of 0.50 which achieved 6.3 s. This result was produced by 100% OPC. From the analysis, it can be seen that the increment in w/c ratio encourages the grout mixtures to become more liquid and to flow easily. This factor will allow more grout slurries to run out more rapidly through the discharge tube of the flow cone tester.

The grout slurries with a w/c ratio of 0.40 to 0.50 shows a higher workability as compared to findings from Zoorob et al. [8], Euco Densit LLC [4], CHKL specification [9], and Koting et al. [11]. This is due to the higher percentage of SP used in the mixture and because of the grout composition. Based on *t*-test analysis, it is found that there is a significant difference in flow values for the three mixes only at 0.35 w/c ratios. For instance, the cement grout containing 95% OPC, 5% SF, 1.5% SP, and 0.35 w/c ratio yielded at value of 32.5 which was significant at the 0.05 level (*P* = 0.0196). The influence of the w/c ratio on the fluidity discussed by Chandra and Björnström [12] stated that the w/c ratio controls the concentration of ions in the pore solution. At a low w/c ratio, the surface of interstitial phases especially of C_3_A and C_4_AF is absorbing the SP; thus very little SP is in the pore solution [12]. However, with an increase in the w/c ratio, more alite hydrates and thereby more Ca^2+^ ions are produced [12]. Lime saturation in the pore solution increases, stifling the hydration process. Subsequently, the fluidity increases [12].

**Figure 2:**
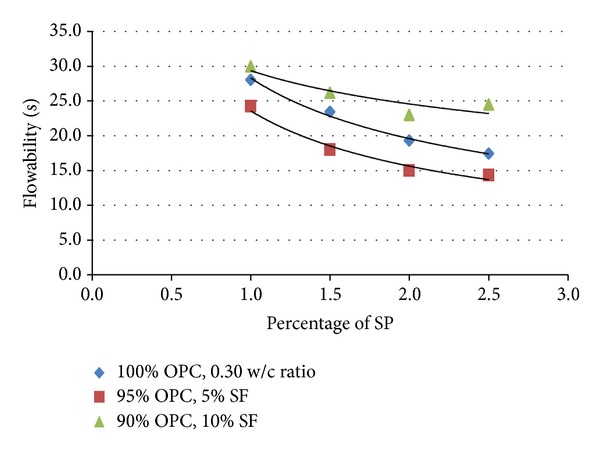
Influence of SP percentages on workability for 100% OPC; 95% OPC, 5% SF; and 90% OPC, 10% SF mixture.

According to Baltazar et al. [13], the fresh grout properties can be improved with the use of additives in the grout composition, including dispersant additives such as superplasticizers (SPs), which cause an important improvement in the grout fluidity and stability through interparticle repulsive forces [13]. The main purpose of adding SP into the grout mixture is to enhance the workability of the cement slurries. The SP provides the possibility of a better dispersion of cement particles, thereby producing a paste of higher fluidity.  Figure 2 shows the relationship between various SP dosages on the workability performance for 100% OPC (GM1); 95% OPC, 5% SF (GM2a); 90% OPC, 10% SF (GM2b); and 0.30 w/c ratios.

Figure 2 shows that the flow values for the three mixes are increased with an increment of SP percentages. The highest workability is produced by 95% OPC, 5% SF, and 2.5% SP which is 14.3 s and the lowest workability is 30 s which is produced by 90% OPC, 10% SF, and 1.0% SP. From *t*-test analysis, there is a significant difference between the two means of flow values for the three mixes only by an increment to the SP percentage from 1.0% to 1.5%. For 95% OPC, 5% SF with 0.30 w/c ratio yielded at value of 13.44 which was significant at the 0.05 level (*P* = 0.0473). The flow also improved by increasing the w/c ratio up to 0.50. At higher w/c ratios, dosage of SP can be decreased up to 0.5%. For 100% OPC, the minimum content of SP used is 0.5% which depends on w/c ratio. The maximum dosage of SP which is sufficient for workability is 2.0% for most of the mixtures. For mixtures containing SF, it is observed that the lower SP dosage which is less than 1.5% causes the cement grout to harden faster and this condition influences the workability. It was also found that at 2.5% SP dosage, samples tend to bleed. “Bleeding” or “water gain” is the tendency for water to rise to the surface of freshly placed concrete [14]. It results from the inability of the constituent materials to hold all the mixing water dispersed throughout the mix [14]. According to Siddique [15], it was observed that mixes incorporating higher silica fume content tended to require higher doses of SP. The high demand of SP with the concrete containing SF was attributed to the very fine particle size of the silica fume that causes some of the SP being absorbed on its surface [15]. In addition, the effectiveness of SP is enhanced by the existence of silica fume, SF. The use of SF affects significantly the properties of fresh concrete [14]. The mix is strongly cohesive and, in consequence; there is very little bleeding or even none [14]. In addition, SP shows a greater effect in SF due to its very fine nature and greater surface area, which increase the water demand [16]. This condition allows the use of high dosage of SP for very low w/c ratio without bleeding or any segregation problems encountered with normal OPC concrete [16].

The use of carboxylic-based SP is also an important criterion in enhancing the workability of grout slurries. This SP is a unique multipurpose admixture particularly suitable for the production of ready mixed concrete and precast concrete [5]. The addition of a carboxylic-based SP resulted in higher workability of the grout compared to when a naphthalene formaldehyde sulphonate based SP was used. The inclusion of a copolymer with a functional sulfonic group and carboxyl group maintains the electrostatic charge on the cement particles and prevents flocculation by adsorption on the surface of cement particles [14]. Instead of controlling the water/cement ratio, the cement dispersion will encourage the development of the grout mixture with higher strength [5]. The use of SPs allows the development of repulsive forces due to the adsorption of the ionic polymers on the surface of the particles. In general, there are two main mechanisms of how SPs disperse binder particles in a suspension: electrostatic repulsion and steric hindrance [13]. The repulsion is caused predominantly by steric hindrance in addition to ionization dispersion forcing; the repulsion arises from the long side chain polymer (SP) which holds the particles far enough so that they cannot come together [13].

### 3.3. Compressive Strength

**Figure 3:**
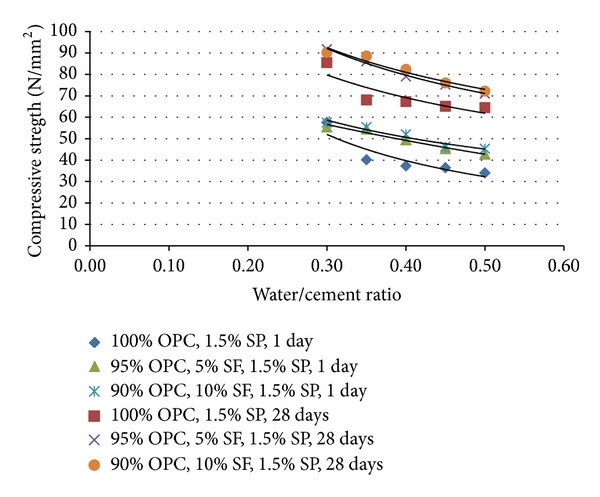
Influence of water/cement ratio on compressive strength at 1 day and 28 days.

The compressive strength performance for all the mixtures was measured at 1, 3, 7, and 28 days. The compressive strength of cement grout is inversely proportional to the increase in w/c ratio; that is, the higher the w/c ratio, the lower the compressive strength, and the lower the w/c ratio, the higher the compressive strength.  Figure 3 shows the relationship between various w/c ratios on compressive strength performance at 1 and 28 days for 100% OPC (GM1); 95% OPC, 5% SF (GM2a); and 90%, 10% SF (GM2b) with 1.5% of SP.

It can be seen that there is a decrease in compressive strength performance, which is related to the increment of the w/c ratio. The compressive strength performance at 1 and 28 days is closely similar for all the mixtures. However, the highest compressive strength at 1 day is 57.6 N/mm^2^ which is produced by 100% OPC, 0.30 w/c ratio, and 1.5% of SP. The cement grout containing 95% OPC, 5% SF, 0.30 w/c ratio, and 1.5% of SP produced the highest compressive strength performance at 28 days (91.7 N/mm^2^). It is observed that (i) at 28 days the grout containing 5% SF was 26% stronger and 10% SF was 30% stronger than the control and (ii) compressive strength for grout containing 5% and 10% SF is almost similar at 28 days.

According to Lee et al. [17], the compressive strengths of specimens with silica fume tended to increase with the increase of the replacement amount and curing age due to a pozzolanic reaction [17]. The results from Lee et al. [17], demonstrate that the addition of silica fume in cement-based composites resulted in a higher compressive strength, a lower absorption, a lower critical pore size, a lower chloride diffusion coefficient, and a lower corrosion rate [17]. This agrees with Neville [14], who noted that the pattern of the relation between compressive strength and the water/cement material ratio is the same for concretes with and without SF but, at the same ratio, concrete with SF has a higher strength. However, the strength probably improves because of the effect of pozzolanic activity or reaction from SF. The three mixes also exhibited a significant decrease in compressive strength in line with the increment of w/c ratio from 0.30 to 0.50. By comparing the results, there is obviously a slight difference in the compressive strength performance, which is produced from this study, CHKL Specification [9], and Zoorob et al. [8]. These are due to different types and properties of materials used, that is, cement, SP, and SF.

In order to obtain adequate strength of cementitious grout, supplementary cement material, that is, silica fume, has been used. Throughout this study, SF was used as a replacement to a small proportion of OPC. The amount of SF used is 5% and 10%. Smaller proportions of silica fume were used because of the very high reactivity of silica fume with calcium hydroxide produced by the hydration of Portland cement [14]. The influence of silica fume on the compressive strength performance for cementitious grouts containing 100% OPC; 95% OPC, 5% SF; and 90% OPC, 10% SF, is shown in Table 2.

From Table 2, the highest compressive strength is 59.7 N/mm^2^ at 1 day and 92.8 N/mm^2^ at 28 days which is produced by 90% OPC, 10% SF, 0.30 w/c ratio, and 2.0% SP. The grout containing 100% OPC, 0.35 w/c ratio, and 2.0% SP produced the lowest strength performance which is 38.3 N/mm^2^ at 1 day and 66.2 N/mm^2^ at 28 days.

A paired-sample *t*-test was carried out to determine whether there is a significant difference between the two means of the compressive strength performance by replacing the grout mixtures with 5% and 10% of SF. Based on *t*-test analysis, it was found that replacing the cementitious grout mixtures with 5% and 10% of SF influenced the compressive strength performance (1 and 28 days) only at 0.35 w/c ratios of 1.5% and 2.0% of SP. As an example, the *t*-test analysis for 1 day strength of 95% OPC, 5% SF, 0.35 w/c ratios, and 1.5% SP yielded at value of 31.67 which was significant even at the 0.05 level (*P* = 0.0201). For the strength after 28 days, this test yielded a *t* value of 45.00 which was significant even at the 0.05 level (*P* = 0.0141). In addition, the influence of increasing SF from 5% to 10% on compressive strength performance at 1 and 28 days was also determined. It was found that increasing the SF content from 5% to 10% did not influence the compressive strength performance at 1 and 28 days. As an example, the *t*-test analysis for 0.30 w/c ratios, 2.0% SP at 1 day strength, yielded at value of 7.29 which was not significant even at the 0.05 level (*P* = 0.0868). For 28 days strength, this test yielded a *t* value of 0.71 which was not significant even at the 0.05 level (*P* = 0.6051).

In summary, we can say that the use of SF as a replacement (5%) in a small proportion of OPC has influenced the compressive strength performance. This is because of the highly reactive fineness of the SF particles, which speeds up the reaction with calcium hydroxide produced by the hydration of Portland cement [18]. The very small particles of silica fume can enter the space between the particles of cement, thus improving the packing [18]. In addition, SF also contributes to the progress of hydration of the latter material [15]. This contribution arises from the extreme fineness of the SF particles, which provide nucleation sites for calcium hydroxide [18]. Thus, early strength development takes place. The contribution of SF to the early strength development (up to about 7 days) is probably through improvement in packing, that is, action as a filler and improvement of the interface zone with the aggregate. In addition, the microfiller effect from SF allows the silica to react rapidly and provides a high early age strength and durability [18]. The efficiency of SF is 3–5 times that of OPC and consequently a vastly improved concrete performance can be obtained [18].

The replacement with adequate amount of SF improves the interface zone with the aggregate. In this case, the replacement of 5% SF is sufficient in order to increase the grout mixtures performance. Thus, the finding of this study is that the contribution of SF to strength increases with an increment in the content of SF in the mix (up to a certain limit). The SP percentages considered in cementitious grout mixture production are 1.5% and 2.0%.

### 3.4. Flexural Strength

The flexural strength test was carried out to determine the tensile strength of cementitious grouts. The flexural strength test is conducted on cementitious grout beams (50 × 50 × 300 mm) according to BS1881: Part 118: 1983 at the ages of 7 and 28 days. The flexural strength for four-point bending setup where the loading span is 1/2 of the support span is calculated as follows:

(1) $$\begin{array}{c} \sigma =\frac{3FL}{{4bd}^{2}}, \end{array}$$

where *F* is the load (force) at the fracture point, *L* is the length of the support (outer) span, *b* is width, and *d* is thickness. The flexural strength performance for selected grout mixtures, which produced sufficient workability and compressive strength, is summarized in Table 3.

The flexural strength performance was investigated after 7 and 28 days. It seems that the use of 5% silica fume influences the flexural strength of cementitious grout mixtures. For further verification, paired-samples *t*-test is carried out. It is found that replacing the cementitious grout mixtures with 5% SF influenced the flexural strength performance at 7 and 28 days. The* t*-test analysis for 95% OPC, 5% SF, 0.30 w/c ratios, and 1.5% SP yielded a *t* value of 53.00 at 7 days and *t* value of 15.12 at 28 days which was significant at the 0.05 level (*P* = 0.0420). Additionally, the grout mixture containing 95% OPC, 5% SF, 2% SP, and 0.30 w/c ratio produced the highest flexural strength at 7 and 28 days. By using an adequate dosage of SP, it will enhance the ability of the grout mixtures to infiltrate into unfilled compacted skeletons under gravitational action. The addition of SP will improve the cement dispersion action thereby producing grouts with higher fluidity. The presence of the long lateral chains which linked to the polymer backbone generates a steric hindrance, which stabilises the cement particles capacity to separate and disperse. This characteristic provides flowable grout with greatly reduced water demand. Instead of controlling the water/cement ratio, the cement dispersion will encourage the development of grout mixture with higher strength.

## 4. Conclusions 

Based on the findings of laboratory investigation on cementitious grout mixtures, the following conclusions can be drawn.

1. High workability and high strength cementitious grouts that can infiltrate into unfilled compacted skeletons can be produced. It can be concluded that the main constituents in producing cementitious grout mixtures are an adequate percentage of cement and pozzolanic materials, that is, silica fume, sufficient amount of SP, and appropriate w/c ratio.
2. It can be said that the appropriate process in producing the cementitious grout mixtures is achieved in this study.
3. The cementitious grouts containing 95% OPC, 5% SF, 0.30 w/c ratio, and 2.0% SP produced sufficient and consistent workability results (in the range of 11 s to 16 s) as compared to other proportions. Therefore, the grout mixture with 5% replacement of SF was considered in producing the final product, which is a cement-bitumen composite (semiflexible pavement surfacing).
4. The results obtained showed that the use of carboxylic-based SP enhances the workability of the grout slurries. It was also found that the sufficient amount of SP for workability requirement is 2.0% for most of the mixtures.
5. It can be concluded that the cementitious grouts containing 95% OPC, 5% silica fume, 0.30 w/c ratio, and 2.0% SP produced sufficient compressive strength which is 57.5 N/mm^2^ at 1 day and 92.5 N/mm^2^ at 28 days. Thus, the replacement of 5% silica fume which is an adequate percentage of SP and w/c ratio was considered in producing the cement-bitumen composites.
6. It can be concluded that the flexural strength performance shows better performance by using 5% replacement of SF. The highest flexural strength is 6.7 N/mm^2^ at 7 days and 9.1 N/mm^2^ at 28 days, which was produced by 95% OPC, 5% SF, 2.0% SP, and 0.30 w/c ratios. This is in line with the compressive strength performance for the same mixture, which produced higher strength at 1 and 28 days. This mixture also produced sufficient workability which is 15 s.

## Figures and Tables

**Table 1:** Mixture proportions.

Grout mixture design	Cement binder (%)		Percentage of SP (%)	Water/cement ratio				
	Ordinary Portland cement (OPC)	Silica Fume (SF)	
Grout mixture 1 (GM1)	100	0	0.5	0.30	0.35	0.40	0.45	0.50
			1.0	0.30	0.35	0.40	0.45	0.50
			1.5	0.30	0.35	0.40	0.45	0.50
			2.0	0.30	0.35	0.40	0.45	0.50
			2.5	0.30	0.35	0.40	0.45	0.50
Grout mixture 2 (GM2a)	95	5	0.5	0.30	0.35	0.40	0.45	0.50
			1.0	0.30	0.35	0.40	0.45	0.50
			1.5	0.30	0.35	0.40	0.45	0.50
			2.0	0.30	0.35	0.40	0.45	0.50
			2.5	0.30	0.35	0.40	0.45	0.50
Grout mixture 2 (GM2b)	90	10	0.5	0.30	0.35	0.40	0.45	0.50
			1.0	0.30	0.35	0.40	0.45	0.50
			1.5	0.30	0.35	0.40	0.45	0.50
			2.0	0.30	0.35	0.40	0.45	0.50
			2.5	0.30	0.35	0.40	0.45	0.50

**Table 2:** Influence of silica fume replacement on the compressive strength performance.

Grout mixture description			Compressive strength (N/mm^2^)	
Grout content	w/c ratio	% of SP	1 day	28 days
100% OPC	0.30	1.5	57.6	85.5
		2.0	59.3	87.2
	0.35	1.5	40.2	68.1
		2.0	38.3	66.2
95% OPC + 5% SF	0.30	1.5	55.4	91.7
		2.0	57.5	92.5
	0.35	1.5	54.4	86.1
		2.0	57.3	87.0
90% OPC + 10% SF	0.30	1.5	57.5	90.2
		2.0	59.7	92.8
	0.35	1.5	55.4	88.6
		2.0	57.1	89.7

**Table 3:** Selected designs and flexural strength of cementitious grout mixtures.

Grout mixture design	Percentage of SP (%)	Water/cement ratio	Flowability (s)	Compressive strength (N/mm^2^)		Flexural strength (N/mm^2^)	
				1 day	28 days	7 days	28 days
95% OPC + 5% SF	1.5	0.30	18.0	55.4	91.7	6.5	7.2
95% OPC + 5% SF	2.0	0.30	15.0	57.5	92.5	6.7	9.1
95% OPC + 5% SF	2.0	0.32	14.2	57.1	91.5	5.8	8.3
100% OPC	2.0	0.30	19.3	59.3	87.2	3.8	6.1
